# Position paper of the EPMA and EFLM: a global vision of the consolidated promotion of an integrative medical approach to advance health care

**DOI:** 10.1186/1878-5085-4-12

**Published:** 2013-05-10

**Authors:** Olga Golubnitschaja, Ian D Watson, Elizabeta Topic, Sverre Sandberg, Maurizio Ferrari, Vincenzo Costigliola

**Affiliations:** 1 European Association for Predictive, Preventive and Personalised Medicine, EPMA, Brussels 1150, Belgium; 2 European Federation of Clinical Chemistry and Laboratory Medicine, Liverpool, L9 7AL, UK; 3 European Society of Predictive Medicine, Milan, 20159, Italy; 4 European Medical Association, Brussels, 1160, Belgium

**Keywords:** Laboratory medicine, Predictive preventive and personalised medicine, Integrative medical record, Health care, Patient in focus, Bioinformatics, Economy, Ethics, Education, Expert recommendations

## Abstract

The authors consider acute problems in the quality and management of medical services challenging health care systems worldwide. This actuality has motivated the representatives of the European Association for Predictive, Preventive and Personalised Medicine and European Federation of Clinical Chemistry and Laboratory Medicine to consider the efforts in promoting an integrative approach based on multidisciplinary expertise to advance health care. The current paper provides a global overview of the problems related to medical services: pandemic scenario in the progression of common chronic diseases, delayed interventional approaches of reactive medicine, poor economy of health care systems, lack of specialised educational programmes, problematic ethical aspects of treatments as well as inadequate communication among professional groups and policymakers. Further, in the form of individual paragraphs, the article presents a consolidated position of the represented European organisations. This position is focused on the patients' needs, expert recommendations for the relevant medical fields and plausible solutions which have a potential to advance health care services if the long-term strategies were to be effectively implemented as proposed here.

## Advancing the health care paradigm

**Figure 1:**
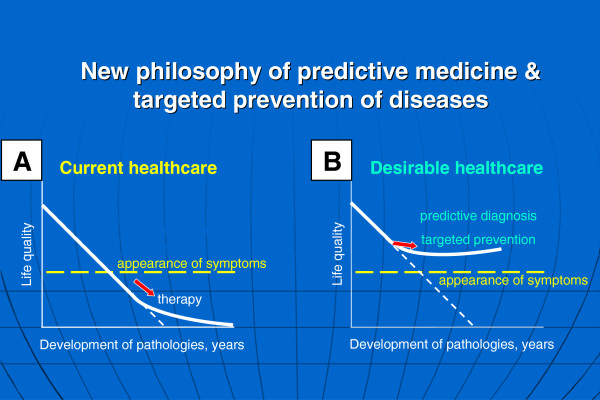
**Current health care approach (A) versus advanced health care approach (B). **The advanced health care approach considers a paradigm change from delayed interventional to predictive, preventive and personalised medicine as the robust platform for optimal medical services; figure is taken from [1].

For many acute and chronic disorders, the current health care outcomes are considered as being inadequate [1]. Current health care practices essentially rely on the emergence of signs and symptoms of human pathologies prior to initiation of interventional modalities as illustrated in Figure 1A. Consequently, despite high costs associated with the absolute majority of currently administrated medical services, long-term morbidity and prognosis may often be poor. This is due to inadequate control of disease manifestations, treatment failure, disease recurrence and the appearance of severe secondary complications contributing to relatively low quality of life for the treated persons and high mortality. Examples of this pessimistic perspective include the pandemic of type 2 diabetes mellitus, neurodegenerative disorders and some types of cancer, which, over the next 10–20 years, associated with the economic situation, could spell disaster for health care systems on a global scale.

A desirable strategy is illustrated in Figure 1B. The major premise of the advanced health care approach is the paradigm change from reactive to predictive medicine, from delayed to preventive and personalised medicine. Since chronic pathologies are generally triggered at the molecular level with consequent symptomatic manifestation of the disease, a laboratory-based detection of pathology-specific molecular patterns would create a well-founded basis for the desirable predictive medical services giving the opportunity for optimal health care. This requires the application of innovative biotechnologies to predict human pathologies, the devising of appropriate and timely preventive strategies and individualised treatment planning.

## Multidisciplinary analytical expertise is essential for accurate diagnosis and optimal clinical decisions: stakeholders in the *board*

Optimal clinical decisions are the result of a multidisciplinary approach that might be viewed as a board of specialists with complementary analytical expertise. Accurate diagnosis and treatments tailored to the person are extremely challenging which drives the development of emerging technologies and harnesses them for the delivery of the requisite medical services. Therefore, the overall task is formulated as the integrative medical approach of the multimodal diagnostics, disease-specific biomarker patterns, individual patient profiles, creation of medical records and treatments tailored to the person [2].

Multimodal diagnostics represents a model-based examination procedure with several levels of examination resulting in extended patient profiles and medical records which obligate inclusion of an interview with the patient / a questionnaire filled in for relevant information on any known pathology, medical imaging, laboratory diagnostics and evaluation of relevant risk factors. For laboratory diagnostics, it is highly recommended to use minimally invasive validated blood tests for the detection of stage-specific molecular patterns at complementary levels of targeted regulation (DNA polymorphisms, transcripts, protein expression, post-translational modification, stage-specific subcellular imaging, shifted enzymatic activity etc.).

## The central role of laboratory medicine as the integrating element in health care services

**Figure 2:**
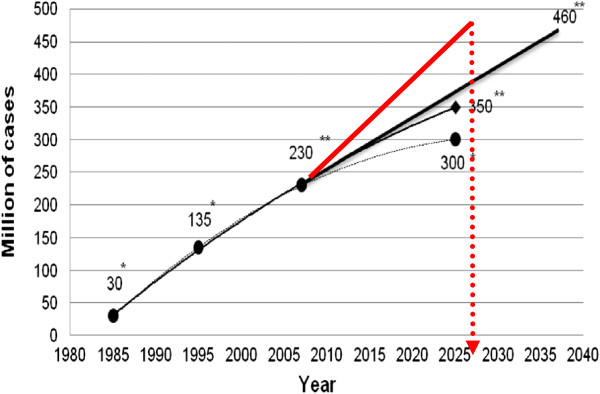
**Worldwide prognosis of the pandemic increase in the incidence of the T2DM. **(*) Estimations as published around the year 2000. (**) Worsening prognosis as published in 2003–2008. Current prognosis is marked in *red colour*; data taken from [3].

It is statistically evidenced that, currently, about 70% of all medical interventions utilise laboratory data. However, this number is expected to rise since, without any exception, all medical fields request robust information from the laboratory to make well-justified clinical decisions. For example, this is the case in (pre-)diabetes care. The pandemic increase in the incidence of type 2 diabetes mellitus (T2DM) year by year is getting worse as demonstrated in Figure 2, and the overall number of diabetic patients is expected to double over the next two decades. Due to the high prevalence of the pathology and severe chronic complications, the application of predictive diagnostics, targeted preventive measures and treatment approaches tailored to the patient are emerging to advance (pre-)diabetes care. This improvement would minimise subsequent complications and associated costs.

The principal difference between the current and highly desirable (pre-)diabetes care is the progress from the unsatisfactory reactive to the predictive and preventive medical services by innovative technologies of *laboratory medicine* applied at the (pre-)clinical disease stages as follows [3]:

1. *First level*. Prediction of the familial predisposition, targeted prevention of T2DM early in childhood.

2. *Second level*. Prediction of early/premature ageing and targeted prevention of T2DM pre-stages.

3. *Third level*. Prediction of secondary complications in the cohort of T2DM patients and creation of treatments tailored to the patient to prevent cardiovascular, neurodegenerative and cancer diseases frequently developed in diabetic patients.

The common onset of T2DM in early adulthood leads to dramatic consequences linked with the early onset of diverse severe complications such as retinopathy, nephropathy, polyneuropathy, dementia, silent ischaemia, ‘diabetic foot,’ endometrium carcinoma and other types of cancer. Chronic degenerative processes affecting vital organs in T2DM usually appear as a ‘domino effect’ in the characteristic sequence as reviewed earlier [4], thereby considering an actively proliferating diabetic retinopathy to be an early indicator of an individual predisposition to the cascade of severe chronic complications developing downstream towards the manifestation of highly proliferative retinopathy. A highly proliferative process in the retina is characterised by pathology-specific molecular patterns which may be detected utilising predictive blood tests some years before the clinical manifestation of the pathology [5]. In this context, the central role of laboratory medicine is to interconnect the relevant medical fields as the integrating element in health care services. In the case of (pre-)diabetes care, laboratory medicine is interrelated to general practitioner/family practice, diabetology, radiology, ophthalmology, endocrinology, gynaecology, sport medicine, professional nutrition etc.

## Shifting of competencies and responsibilities in the laboratory-clinician interface

Shifting the role of laboratory from the ‘passive performing’ to the ‘active advising’ is the next paradigm change in health care. This reconsideration of the laboratory-clinician interface might significantly advance the quality of current medical services, although the implementation of this approach across countries should be adapted to local conditions. Well-known motivating actualities for the reconsideration are, for example, so-called *within-individual* variations as well as *grey-zone* results, when individual laboratory parameters range between ‘healthy’ and ‘unhealthy’ zones and any interpretation is rather problematic.

Therefore, recommendations by the laboratory to assist clinical practice are highly desirable [6]. This assistance ranges from advising on the necessity for additional tests to the dynamic analysis of the targets. Additional tests should be considered from the viewpoint of their reasonability, in order to reach an accurate and realistic health-related data interpretation for the individual. The analysis of dynamic changes of the target is essential to evaluate potential health impacts such as an individual predisposition to the disease and/or a predictive diagnosis before a clinical manifestation of symptoms. Laboratory value-added investigation and interpretation should be obligatory in creating an advanced functional relationship between laboratory medicine and clinicians together as the responsible decision-makers [7].

## Predictive/prognostic laboratory tests as the robust platform for targeted prevention and treatments tailored to the person

The following actualities motivate the introduction of predictive and prognostic laboratory tests into daily medical services:

• Delayed intervention

• Untargeted medication

• Overdosed and poisoned patients

 Ineffective treatments

Current deficits in medical services such as missing toxicological examination of the prescribed medication and ethically problematic delayed and inadequate treatments require new strategies for a targeted prevention and more effective treatments tailored to the person [8]. Validated predictive and prognostic laboratory tests provide new perspectives on how to improve diagnostic and prognostic performances. In order to create a robust investigative platform, the utilisation of appropriate laboratory tests should be mandated, which means that an extensive collaboration between laboratory professional organisations on one side and policymakers on the other side as soon as practicable to intensify the process.

## Accuracy, sensitivity, specificity and unification of analytical tests: status quo and future perspectives

The above requirements lead to obligatory prioritising of the improved analytical tests which might be utilised in order to advance medical services today, in the near future but certainly in the long-term perspective. The undoubted criteria are the following:

1. Diagnostic sensitivity,

2. Diagnostic specificity,

3. Clinical utility and

4. Unification.

Traceability of a measurand to a certified reference material and measurements validated by a definitive analytical method leads to the ‘unification’ of a given laboratory test. There is the evident necessity to enable globally comparable investigative, diagnostic and prognostic strategies to be reliably implemented. This requires regulatory oversight and meaningful engagement with *in vitro* diagnostic providers. The globalisation of markets and laboratory-related business requires the comparability, or the unification, of laboratory test values. The mobility of both patients and health care professionals as well as the increasing global data flow require such comparability [9]. Hence, the unification of laboratory tests should be placed at the top of the list of corresponding adapting measures.

## Disease monitoring, self-monitoring and distanced monitoring

Current trends in Europe demonstrate dramatic demographic shifts in favour of elderly subpopulations followed by the consequent alterations in the overall profile of the patient cohorts. With *type 2 diabetes mellitus*, pandemic and its severe secondary complication such as *stroke*, 50% of people after 85 years of age affected by *Alzheimer's disease* (also diagnosed as *type 3 diabetes*) and neurodegenerative eye diseases with leading causes of blindness, *diabetic retinopathy* and estimated 70 million *glaucoma* patients worldwide, millions of patients with *Parkinson’s disease*, *multiple sclerosis, epilepsy* and *dementia* in the elderly—altogether dramatically affect life quality and social and economic indexes of populations around the globe. According to the current prognoses, it is predicted that neurodegenerative disorders will achieve more than 30% of the global disease burden till 2025 [10]. These realities require new strategies in health care in terms of long-term monitoring of chronically diseased patients with a potential duration of some years to several decades of life [11]. A new generation of ‘point-of-care’ monitoring devices is required. These mobile health technologies must enable both the remote management of the analytical process and the active engagement of laboratory professionals at the clinical level.

## Promotion of the concepts of ‘participatory’ medicine: improving health knowledge and health language in the general population and collaboration with organised patient groups

‘Nothing about me without me’ is an excellent slogan of the Society of Participatory Medicine [12] which certainly should be broadly accepted by advanced medical services. A number of independent evidence-based studies have demonstrated that the efficacy of treatments strongly depends on the level of harmony in ‘doctor-patient’ collaboration. However, the critical question remains: how can a common language that is understandable for the professionals in the health care industry and patients be created? The only solution is high didactic quality educational measures aiming at significant improvements of health knowledge and health language in the general population. Unfortunately, information retrieved from the internet is frequently of poor professional quality providing controversial data that confuse the understanding and slow down the learning process of laymen. People need to be advised of reliable information sources that are well adapted to a corresponding level of understanding (categories of children, youth and adults) and concrete interests of subpopulations (level of education, groups of professionals, patient cohorts). In the field of education, laboratory medicine may play a leading role providing up-to-date information that is accessible to the layman on laboratory tests and their interpretation for individual health and disease conditions. A professional version will enable for a detailed knowledge about bioactive molecules, enzymatic reactions, molecular and cellular processes which underlie the pathomechanisms of individual predispositions and pathologies as well as medical treatments. These innovations along the tight collaboration of organised patient groups are one of the strongest instruments of a more effective knowledge promotion [13].

## Are ‘more laboratory tests’ equal to ‘better life quality’? Economy and ethics of personalised medicine—patient in focus

The well-known problem of the intensity of medical services in general and a reasonable number of laboratory tests to be performed in individual cases in particular should be considered in the context of the economy of personalised medicine (how much?) on one side and the ethical issue (is it good enough?) on the other side. However, the middle point between both factors under consideration should be an obligatory recognition of the long-term welfare of the patient [7]. An optimal evaluation of the individual contributing factors enables ethically correct and economically justified clinical decisions. However, currently, no one is able to estimate all the genetic, epigenetic, lifestyle-dependent, environmental, nutritional factors in an individual to provide the level of recommendations and adequate prognostication.

## Creation of innovative medical records: a ‘happy marriage’ with integrative bioinformatics in the near future

How can integrative analytical data in the above-delineated context and for the listed purposes be treated? A creation of innovative medical records should be considered as a priority for scientific programmes of multidisciplinary character. Indeed, corresponding budgets are currently under extensive discussion for the new European programme in preparation for personalised medicine, the ‘Horizon 2020’ [14]. An integrative bioinformatics is considered as the powerful tool to fulfil this highly ambitious task since it becomes essential to the following:

• Gathering of complex data received from emerging technologies, such as medical imaging, pharmacogenetics, clinical ‘-omics’, pathology-specific molecular patters, disease modelling and individual patient profiles

• Undertaking both retrospective and prospective analyses of biodata, information and knowledge related to the (epi-) genome and its links to disease for translational medicine

• Providing help with information and knowledge to advance health-related sciences and to make health care services more reliable

• Analysing complex technological inputs for making optimal clinical decisions

• Learning from what has happened at the individual, process and health system levels to promote the integrative approach by predictive, preventive and personalised medicine (PPPM)

• Creating patient records and securing safety treatment of patient databases

• Promoting standardisation in health care

• Presenting integrated data innovatively to enhance comprehension

Creation of innovative medical records might be a strong driver for standardising communications in the broader health-related scientific community and health care industry [14,15].

## Progressing from ‘disease care’ to ‘health care’: ‘well-being’ concept—what is beyond the issue?

The evident pandemic of common chronic diseases, the consequences of a reactive approach and hence poor economy of disease care as well as dramatic social and ethical problems that resulted from delayed medical services necessitate experts to reconsider the current paradigm of reactive medicine in favour of the macroeconomic, more efficient predictive and targeted preventive measures. This means that there is a mandated progressive implementation of a new philosophy of the well-being concept tailored to the person (age, gender, socio-economic status, individual predispositions, patient-specific profile etc.) as a carefully elaborated spectrum of measures for professional care that promotes the mental and physical health of an individual [8].

Which among the new services might be introduced in order to promote the implementation of the well-being concept? It will be based on the individualisation of analytical evidence of individuals' interaction with their environment and the impact of avoiding or mitigating its environmental exposure on wellness. Sufficiently, better individual outcomes are expected with respect to less morbidity and a better quality of life. Herewith, laboratory medicine is considered an essential contributor to such assessments, leading to a hope for both improved health economy and ethical standards [16,17].

## Towards the question of who may benefit from patient records: sensitive ethical aspects behind PPPM

From what was mentioned previously, it is getting clear that the innovative technologies of multidisciplinary analysis summarised by comprehensive patient records provide the complete information about current pathology-specific profiles and potential predispositions of the person. This reality creates a number of socio-economic questions and concomitant ethical issues such as the following:

• Who is allowed access to individual records in the database?

• Is the current technological platform robust enough to guarantee authorised access only and to secure the rights of the patient and specialised PPPM centres?

• Who is authorised to interpret these individual records?

• What are the expected and potential long-term consequences of the record for the patient: health benefits versus potential danger of data misuse?

• How well are the patient's interests and specialised centre defended for optimal PPPM performance in the society? Is the juristic platform robust enough or does it need to be extended?

Robust information governance of the database and high quality of ethical standards are the prerequisites for the successful implementation of PPPM in health care systems [18].

## Biobanking: meaning, optimal organisation and perspectives in a global scale

*Ethically correct and technologically excellent bio-preservation and biobanking are central activities in the field of PPPM.* This was formulated in the *EPMA* White Paper in 2012 [13].

Internationally valid biobanking is happening now. For that, a list of essential requirements should be functionally satisfied. Competencies should be harmonised among the main stakeholders, namely patients, scientific community and health care providers which includes commercial companies interested in *in vitro* diagnostics, development of new biomarkers and novel drug targets. The following unsolved questions remain:

• How can local and international barriers be broken down to unify international biopreservation technologies, sample collections and databases?

• Who carries the role to promote in the process of biobanking internationalisation?

• Who should cover the costs of the internationally accessible systems?

• Who is allowed to legally use these international biobanks once they are created?

• Who could be nominated as the end user of the data collected?

Currently, biobanking is facing major viability challenges. As for individual types of biological material (tissue samples, blood samples, DNA, RNA, proteins, metabolites etc.), national laws vary for local instructions and legal approaches and how samples may be collected, stored, retrieved and tested. In plenty of cases, this may be quite restrictive and incompatible with rules that are legal for other countries. The analytical quality of collections is frequently compromised as the storage condition for the samples is changing. Donation of samples to a biobank not only requires anonymity but also strict control over record linkage and access. Sample collections with a permission of specialised ethical commission should be hosted by viable academic units and health facilities with a proven record of research and should not be exploited for commercial gain but for the common good. Disease-focused collections require acquired samples to be retrospectively valid for a development of novel biomarkers. Such biomarkers need to have a demonstrated clinical utility prior to their introduction in medical services. For a disease-specific biobanking, the disease needs to have been well characterised for each patient, with immaculate record-keeping, to enable to draw conclusions on the new markers.

These critical problems may be optimally solved by relevant professional groups with complementary expertise such as the European Federation of Clinical Chemistry and Laboratory Medicine, EPMA and ESBB followed by adequate decisions of policymakers resulting in the creation of a robust juristic platform. An international biobanking, if designed as being reliable for advanced health care services, needs an adequate juristic platform that considers the following interests of all parties involved in the research and business:

1. Donors of biological materials,

2. Clinicians providing patient records,

3. Laboratory units collecting the samples,

4. Storage units,

5. Research analysing the collections and creating new diagnostic and drug targets,

6. Patenting institutions,

7. Health care industry,

8. Patient cohorts,

9. Groups at risk,

10. General population and

11. Educators.

## Forcing the practical application of novel molecular targets, specific biomarkers and more efficient laboratory tests

**Figure 3:**
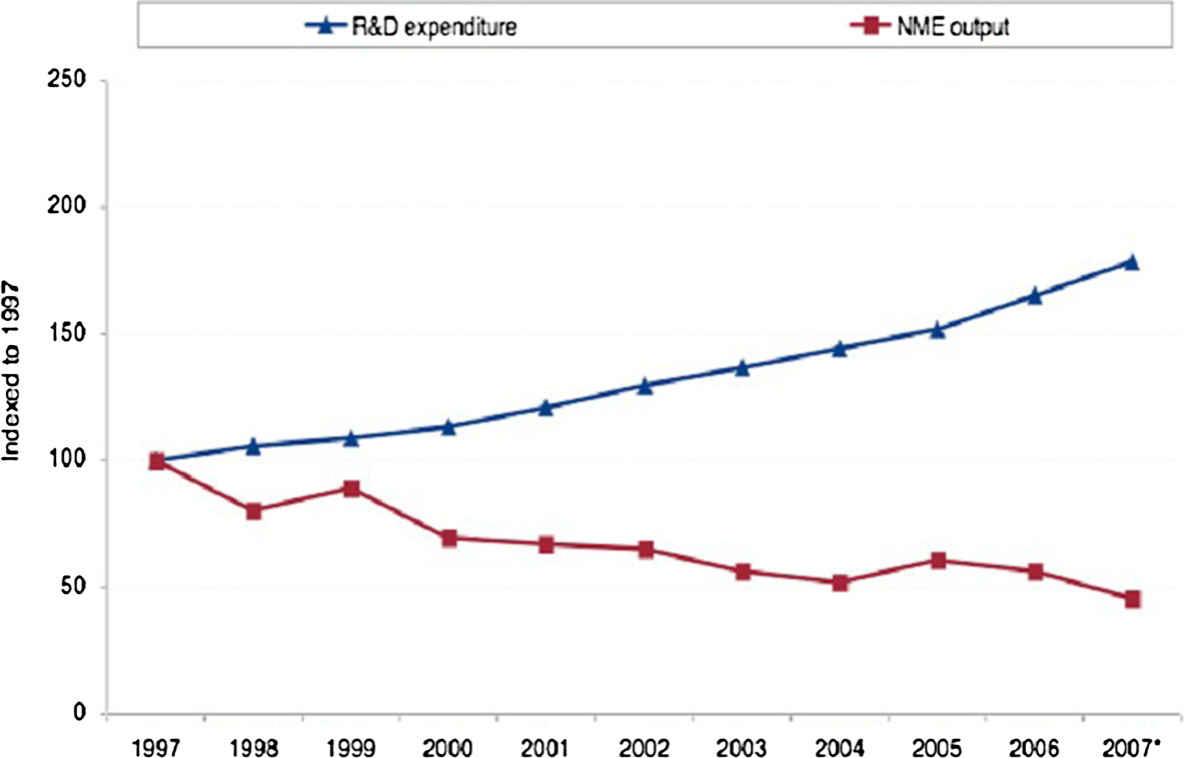
**R&D expenditure versus new molecular targets as observed in industrial developments in 1997–2007 [20]. **NME, new molecule entity.

Current calculations demonstrate a long-term trend of the dramatic crisis in the creation of clinically useful molecular entities (biomarkers, drug targets etc.). Although the graph presented in Figure 3 holds for the original producers of potential molecular targets, within the horizon of 10–15 years, the same problems may be predicted for and confronted by the generic companies. Further, an innovation in *in vitro* diagnostic testing is hampered by a lack of funding for studies aiming at the effective validation of the clinical utility of novel biomarkers. Not only is there a need for meaningful health technology assessments but also a need to remove supplanted markers to minimise financial wastage that could otherwise be better spent. Consequently, a deep analysis of currently unfavourable situations followed by the creation of potential ways of how to shorten the time span between a discovery of new molecular targets and their effective utilisation in daily practice is the strategic point of highest priority [19]. Up to this point, expert recommendations have been summarised in the *EPMA* White Paper in 2012 [13]. The proposed measures include the following:

• Emerging technologies in population screening (faster, more precise, cheaper)

• Set-up of pathology-specific biomarker patterns instead of ineffective single molecules

• Non- or minimally invasive diagnostic tools (saliva, urea, blood etc.)

• Effective promotion of validation studies

• Transparent schemes for functional contacts between researchers who discovered new targets and implementing industrial partners

• Dramatic improvements in national and international patenting systems

An effective implementation of the above-listed measures may favour more flexible small- and middle-sized industrial companies in the competitive process of PPPM-related innovation.

Providing adequate budgets, a strategic reconsideration of current investments should be performed. Hence, having clinical trials that are costly and usually recreated for every new marker is a typical example of wasting money. In contrast, the creation of consolidated laboratory networks of equivalent standards to accelerate clinical validation across the countries of the European Union represents the optimal way on how to generate sufficient professional power in an economically reasonable approach. Therefore, further consideration of these two crucial steps in the overall process to advance health care is essential:

A. Consolidation of professional efforts and

B. Standardisation of healthcare services in the Europe.

See the following sections for details.

## Consolidation of professional groups involved in health care sciences and services

### Status quo

Currently, any multidisciplinary meeting and collaboration of professional groups related to health care usually are results of an enthusiastic initiative of single groups who realise that a multidisciplinary consolidation is the only way on how to advance actual knowledge and services.

### Improved approach

Consolidation of professional groups involved in health care sciences and services is a multifunctional task which should be performed at several levels of activities:

• Mandated optimal set-up of multidisciplinary stakeholders in diagnostics and in the prevention and treatment of individual pathologies

• Regular meetings of stakeholders with multidisciplinary expertise motivated and regulated by health care-related national and international programmes

• Multifunctional education as an obligation for related professional groups

• Creation of optimal economic conditions motivating a multidisciplinary expertise to be applied in health care sciences and services

## Standardisation of health care services in Europe: how to proceed

This is a strategic issue for policymakers after detailed consultations with health care professionals. In order to effectively promote medical services, we need much more harmonisation as has been currently observe in health care across Europe. The process of harmonisation requires clear focuses and should result in reasonable standards introduced for comparable/analogous functional elements and systems. All of the existing systems should be carefully analysed at the European level before corresponding standards may be elaborated and introduced in daily medical practice. European standards have been recommended by experts.

The European standards recommended for *analytical systems* are as follows:

• Consistent standards of laboratory practice

• Conformance with all relevant ISO/CEN standards

• Equivalent units of measurement

• Traceability of the measurand

• Equal quality of measurements

• Equal reference systems

• Equal evaluation systems

The European standards recommended for *the European electronic patient record* are as follows:

• Mandated multidisciplinary examination approach

• Unified communication protocols for data exchange between different information systems in health care

• Consolidated algorithms

• Standardised and complex interpretation of analytical results

It is obvious that these listed measures are tasks superior to the national level. This may be effectively achieved if corresponding European resources will be adequately mobilised, including consolidated professional efforts and dedicated budgets.

## Professional education: meaning and instruments

Professional groups involved in health care-relevant research and services realise step by step that they speak ‘different professional languages’ that are less understandable for other related branches. Consequently, great discoveries made/innovations triggered by one professional group are frequently underestimated, or even not valued at all, by other experts, thus resulting in delays in the implementation of novel developments in medical branches. Therefore, we need to develop a new culture among experts in order to promote the multidisciplinary character of predictive, preventive and personalised medicine and concomitantly to advance currently deficient health care services. Herewith, the authors present a consolidated position to the point made as follows: the innovative PPPM-related educational programmes for professionals should be prioritised in the *Common Strategic Framework* (also called as the New European Framework Programme Horizon 2020) as well as by other global and topic-relevant national programmes [14].

In order to promote innovative educational programmes, the following worldwide pioneer initiatives have been developed to create didactic materials for traditional and innovative medical fields: 

1. Since 2010, *The EPMA Journal* (open access, PubMed indexed) regularly updates both needs and achievements in the field of PPPM applied to common and rare pathologies [21]. The following topics are treated by the journal:

• Health care overview in European countries and worldwide

• PPPM applied to common diseases such as diabetes mellitus, cardiovascular disease, cancer and neurodegenerative disorders

• PPPM in rare diseases

• PPPM in dentistry

• Frequent syndromes and groups at risk

•Reproductive medicine

• PPPM in pregnancy, neonatology and paediatrics

• Body culture, individualised exercise training, nutrition and doping control

• Evidence-based traditional and alternative medicine

• Integrative bioinformatics, patient records and disease modelling

•Treatments tailored to the person

• Validation, standardisation and economic, ethical, legal, social and other relevant aspects of PPPM

2. In 2012, Springer has launched a new book series entitles *Advances in Predictive, Preventive and Personalised Medicine* in collaboration with the European Association for Predictive, Preventive and Personalised Medicine [22]. The first volume in the series, *Healthcare Overview: New Perspectives* (edited by V. Costigliola) has been released in October 2012. Approximately three books will be published each year.

The book series *Advances in Predictive, Preventive and Personalised Medicine* provides an overview of multidisciplinary aspects of advanced biomedical approaches and innovative technologies in health care. Topics focus on cost-effective management tailored to the person in health and disease, and innovative strategies for standardisation of health care services. The book series also includes new guidelines for medical ethics, innovative approaches to early and predictive diagnostics, targeted prevention in healthy individuals, and health care economy and marketing. Innovative predictive, preventive and personalised medicine is emerging as the focal point of efforts in health care aimed at curbing the prevalence of common (diabetes mellitus, cardiovascular diseases, chronic respiratory diseases and cancer) and rare diseases. This new book series is intended to serve as a reference source for researchers and the health care industry with special emphasis on health promotion in the general population.

## Political regulations to advance health care systems

Through the support of the Alexander von Humboldt Foundation (Prof. Dr. Lotta Salminen of Finland and the host supervisor Prof. Dr. Olga Golubnitschaja of Germany were awarded fellowship), in the years 2012–2013, EPMA has performed a specialised project focused on the collaboration among PPPM-related professional groups and policymakers. This project is aimed to identify problems and deficits in health care of common and pandemic chronic diseases such as type 2 diabetes mellitus. Interviews performed with the experts from all countries of the European Union, Israel, Russia and Turkey resulted in the conclusion that one of the common deficits is missing communication and collaboration between health care professionals and decision-makers. Moreover, there are difficulties in accessing national regulatory bodies to enhance the message of patient-focused health care. This evidence demonstrates that decisions in the health care sector are generally made without consideration of accumulated professional knowledge. This may explain the lack of sufficient efficacy currently observed in health care systems. The completed report with the collected data that resulted from this project is currently prepared for publication in *The EPMA Journal* (2013).

In contrast, if well established, the collaboration with governmental institutions may lead to the crucial reconsideration of current national and European guidelines for health care-relevant research activities and medical services. There are following emerging issues to be reconsidered in health care:

1. Promoting research fields focused on the patient needs

2. Mandating a paradigm shift from reactive to predictive and preventive medicine

3. Creating specialised state budgets to target preventive measures against pandemic incidence of common chronic diseases

4. Forcing an effective implementation of innovative predictive diagnostics

5. Creating new educational programmes for professionals to implement integrative medical services in predictive, preventive and personalised medicine

6. Creating educational measures to increase the understanding of innovative diagnostic technologies and targeted preventive measures in the general population

7. Creating new economic models to motivate health responsibility.

As health care is subject to national subsidiarity, there are substantial variations in the level of provision in different countries. However, corresponding standards might be introduced at the European level to promote inter-European mobility and to avoid economic discrepancies and conflict of interest [23].

## Summary

An integrative medical approach to advance health care services is a multidisciplinary task that covers well-harmonised international efforts in research, business and politics. In this article, a consolidated position of the European Federation of Clinical Chemistry and Laboratory Medicine and European Association for Predictive, Preventive and Personalised Medicine is presented. The authors have identified the priorities which might effectively navigate the overall process of the professional consolidation and quality promotion in the field. In order to realise the proposed initiatives, the undersigned organisations consider the creation of the working group of experts as the follow-up action to work out common strategies and to elaborate corresponding structures, scientific proposals and recommendations for the European standards.

## Competing interests

The authors declare that they have no competing interests.

## Authors' contributions

OG and IDW drafted the manuscript. ET, SS, MF and VC completed and critically revised the manuscript. All authors read and approved the manuscript.

## Authors’ information

European Association for Predictive, Preventive and Personalised Medicine, EPMA, Brussels 1150, Belgium, http://www.epmanet.eu. European Federation of Clinical Chemistry and Laboratory Medicine, Liverpool L9 7AL, UK, http://efcclm.eu. European Society of Predictive Medicine, Milan 20159, Italy, http://www.euspm.org. European Medical Association, Brussels 1160, Belgium, http://www.emanet.org.
